# CpG-island methylation of the ER promoter in colorectal cancer: analysis of micrometastases in lymph nodes from UICC stage I and II patients

**DOI:** 10.1038/sj.bjc.6604859

**Published:** 2009-01-13

**Authors:** J Harder, V Engelstaedter, H Usadel, S Lassmann, M Werner, P Baier, F Otto, M Varbanova, E Schaeffner, M Olschewski, H E Blum, O G Opitz

**Affiliations:** 1 Department of Medicine II, University Medical Center, Freiburg, Germany; 2 Institute of Pathology, University Medical Center, Freiburg, Germany; 3 Department of Surgery, University Medical Center, Freiburg, Germany; 4 Department of Medicine I, University Medical Center, Freiburg, Germany; 5 Tumorzentrum ZeTuP, Center for Tumor Detection, Treatment and Prevention, St Gallen, Switzerland; 6 Department of Medicine, Charite, Campus Virchow, Berlin, Germany; 7 Department of Medical Biometry and Statistics, University of Freiburg, Freiburg, Germany; 8 Tumorzentrum Ludwig Heilmeyer – Comprehensive Cancer Center, Freiburg, Germany

**Keywords:** molecular marker, colorectal cancer, micrometastases

## Abstract

Patients with UICC stage II colorectal cancer (CRC) have a risk of approximately 20% to develop disease recurrence after tumour resection. The presence and significance of micrometastases for locoregional recurrence in these patients lacking histopathological lymph node involvement on routine stained HE sections is undefined. Oestrogen receptor (ER) promoter methylation has earlier been identified in CRC. Therefore, we evaluated the methylation status of the ER promoter in lymph nodes from 49 patients with CRC UICC stage I and II as a molecular marker of micrometastases and predictor of local recurrence. DNA from 574 paraffin-embedded lymph nodes was isolated and treated with bisulphite. For the detection of methylated ER promoter sequences, quantitative real-time methylation-specific PCR was used. Of the 49 patients tested, 15 (31%) had ER methylation-positive lymph nodes. Thirteen of those (86%) remained disease free and two (14%) developed local recurrence. In the resected lymph nodes of 34 of the 49 patients (69%), no ER promoter methylation could be detected and none of these patients experienced a local relapse. The methylation status of the ER promoter in lymph nodes of UICC stage I and II CRC patients may be a useful marker for the identification of patients at a high risk for local recurrence.

Colorectal cancer (CRC) is the third most common cancer in western countries with an incidence of 45–60 per 100 000 per year in Europe and the United States (Landis *et al*, 1998). In CRC, the histopathological lymph node status at the time of surgery is one of the main prognostic factors. Adjuvant chemotherapy has been shown to improve disease-free survival by 20–30% in patients after curative resection for colon cancer metastatic to regional lymph nodes (UICC stage III; Dukes C). In contrast, in UICC stage II disease (T3–4, N0, M0; Dukes stage B), only a minimal survival benefit can be achieved with adjuvant chemotherapy. Therefore, patients with UICC stage II CRC are treated with adjuvant chemotherapy only in the presence of additional negative prognostic factors indicating a high risk for relapse (e.g. undifferentiated tumours, tumour perforation). However, approximately 20% of the UICC stage II patients develop local recurrence or distant metastases and would potentially benefit from adjuvant chemotherapy. At present, these patients cannot be identified at the time of surgery.

The presence and prognostic relevance of lymph node micrometastases in CRC patients without routine histopathological evidence of lymph node involvement by standard criteria is unclear. Several molecular markers including CEA, CK, MUC2 and *p53* have been used to detect micrometastases in regional lymph nodes. However, to date, these markers investigated are unreliable due to low sensitivity and/or low specificity (Sanchez-Cespedes *et al*, 1999; Noura *et al*, 2002b; Rosenberg *et al*, 2002; Ho *et al*, 2004; Lee *et al*, 2006). Depending on the respective marker and method used, micrometastases were detected in 20–100% of resected histologically negative lymph nodes of CRC patients with varying results regarding their prognostic value (Futamura *et al*, 1998). This wide range of positive results for micrometastases in resected lymph nodes is clearly influenced by differences in the specificity and sensitivity of the candidate markers and detection methods used. In addition, it raises the question of the clinical significance of single tumour cells for disease recurrence.

Issa *et al* (1994) first described the methylation of CpG (cytosine phospho guanine) islands in the promoter of the oestrogen receptor (ER) in CRC. Non-malignant tissues from thyroid, breast, lung, cervix and prostate were examined for the presence of ER promoter methylation and found to be negative (Esteller *et al*, 2001). In contrast, ER promoter methylation seems to play a role in the early steps of carcinogenesis in several tumour sites including lymphoma, oesophageal cancer and CRC (Issa *et al*, 1994, 1996; Eads *et al*, 2000), being present in almost 100% of primary colorectal tumours. In addition to its potential role in colon carcinogenesis, CpG island methylation of the ER promoter might therefore be a useful marker for molecular detection of micrometastases in lymph nodes of CRC patients.

In addition to micrometastases, genetic subtypes or distinct molecular signatures of the primary tumour have been linked to the probability of local recurrence (Lenz *et al*, 1998; Gryfe *et al*, 2000; Kim *et al*, 2007). Kim *et al* (2007) showed a higher recurrence-free survival for patients with microsatellite-instable (MSI+) tumours and Gryfe *et al* (2000) could show that MSI+ tumours have a decreased likelihood of metastasising to regional lymph nodes. Other molecular markers such as *p53* overexpression and thymidylate synthase have also been linked to higher recurrence rates (Lenz *et al*, 1998). Except for one report, promoter methylation other than hMLH1 and hMSH2, which then leads to MSI, has not been linked to disease recurrence (de Maat *et al*, 2008). MSI+ tumours are more frequent in the right colon (Kim *et al*, 2007) and define one subtype of CRC (Jass, 2007). Nevertheless, ER promoter methylation is more frequent in the left colon and rectum at least in normal tissue independent of the location of CRC (Issa *et al*, 1994).

During surgical resection of CRC, draining lymph nodes are removed together with the primary tumour. The number of metastatic lymph nodes, and also the absolute number of resected lymph nodes or as shown in recent reports the lymph node ratio of metastatic to non-metastatic lymph nodes, predicts the likelihood of disease recurrence in CRC stage III patients. This might be explained by the fact that resected metastatic lymph nodes drain into lymph node areas, which are not resected by the surgeon (Yagci *et al*, 2007). As micrometastases are thought to cause local recurrence through this route, the detection of micrometastases by real-time methylation-specific PCR (rt-MSP) in resected lymph nodes could be a tool to predict local disease recurrence caused by single tumour cells remaining outside the resected area.

Therefore, the aim of this study was to define the value of ER promoter methylation, detected by rt-MSP in resected lymph nodes from patients with CRC UICC stage I and II, as a predictor of local disease recurrence.

## Materials and methods

### Study population

**Figure 1:**
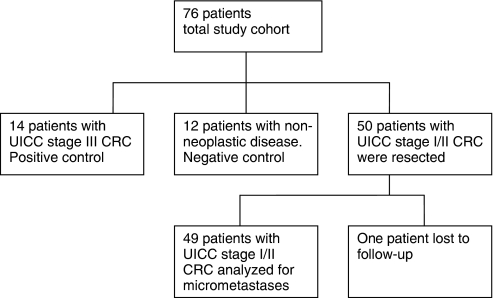
The whole study population comprised 76 patients. Fourteen patients were in the positive and 12 in the negative control group. Fifty patients had been resected for stage I or II CRC. As one patient was lost to follow-up, 49 patients were analysed by ER promoter rt-MSP.

A total of 76 patients were examined in this study (Figure 1). As positive control, primary tumours and microscopically proven metastatic lymph nodes from 14 patients with CRC UICC stage III disease (any T, N1–2, M0) were analysed. As negative control, served lymph nodes from 12 patients obtained from colon resection for non-neoplastic diseases, for example ileus and ulcerative colitis. Fifty consecutive patients with CRC UICC stage I or II disease underwent complete tumour resection at the Department of General and Visceral Surgery at the University Medical Center Freiburg, Germany, between January 2000 and December 2002. One patient was lost to follow-up, therefore 49 patients were analysed for the presence of micrometastases. The latter only included patients with completely (R0) resected sporadic CRC, and histopathologically classified pN0 (negative) lymph nodes were analysed in this retrospective study. The patients with rectal cancer did not have neoadjuvant therapy. Microsatellite status was assessed earlier in 27 of these 49 primary CRCs (Gerlach *et al*, 2006). In all, 7 out of 27 (26%) of the cases were MSI positive and 20 out of 27 (74%) were MSI negative.

### Lymph node processing and DNA extraction

Serial sections of surgically resected lymph nodes were analysed after having been completely processed by formalin fixation, paraffin embedding and routine diagnostics of histopathological TNM classification, for the presence of micrometastases by rt-MSP. In total, 574 lymph nodes (mean 9.8 per patient, range 1–29) were available for analysis. Case-specific clinicopathological and experimental parameters, including the number of lymph nodes analysed by rt-MSP and clinical follow-up, are given in Table 2.

Six serial sections were used for DNA isolation (6 × 10 *μ*m) and one section (3 *μ*m) for HE staining. The lymph nodes were manually microdissected and pooled for DNA isolation to yield one sample per patient. Tissue sections were deparaffinised in xylene and rehydrated using ethanol in decreasing concentrations. Rehydrated tissue sections were digested using proteinase K dissolved in TE buffer diluted at 1 : 10 in distilled water. Genomic DNA was isolated using a commercially available DNA extraction kit (DNeasy®; Qiagen, Hilden, Germany). The DNA concentration of each patient sample was measured (Nanodrop®; PeqLab, Erlangen, Germany) with a mean concentration of 157 ng *μ*l^−1^ (±82 ng *μ*l^−1^). Two micrograms of each DNA sample were treated with sodium bisulphite converting unmethylated cytosine to uracil and leaving methylated cytosine intact, as described by Herman *et al* (1996). After bisulphite modification, the DNA was purified and eluted in 20 *μ*l H_2_O.

### ER promoter analysis

To define the most suitable methylated ER promoter region, we first established a conventional MSP. Lapidus *et al* (1998) divided the CpG island of the ER promoter into six regions (ER1–ER6) and tested them for methylation and its functional relevance regarding gene expression in breast cancer cell lines. Comparison of the different primer sets showed the region ER4 of the CpG island to be most specific. This region was identical to the region analysed by Issa *et al* (1994) for its functional relevance. The primer and probe of the more sensitive rt-MSP used to analyse the study population as well as the positive and negative control group also cover this region and were published recently (Eads *et al*, 2000). Overall, the primer sequences used are as follows: MSP forward primer, 5′-cgagctggagcccctgaaccgtcc-3′ and MSP reverse primer, 5′-cggccgccgccaacgcgcag-3′. For rt-MSP, the following oligonucleotides were used: forward primer, 5′-ggcgttcgttttgggattg-3′; reverse primer, 5′-gccgacacgcgaactctaa-3′; TaqMan® probe, FAM 5′-cgataaaaccgaacgacccgacga-3′ TAMRA. PCR primers were obtained by Genescan (Freiburg, Germany). The probe was custom synthesised by Applied Biosystems (Foster City, CA, USA). Concentrations were 9 *μ*M for the forward, 3 *μ*M for the reverse primer and 2.5 *μ*M for the probe.

Two microlitres of the eluate containing the bisulphite-treated DNA has been used for each rt-MSP. Amplifications were carried out in 96-well plates in a 7000 Sequence detector (Applied Biosystems) in triplets using Universal Mastermix® (Applied Biosystems). The reaction volume was 25 *μ*l. Thermal cycling was initiated by denaturation at 95°C for 10 min. The PCR profile was 95°C for 15 s and 60°C for 1 min for a total of 50 cycles. Each plate contained patient samples and multiple water blanks as well as positive (MDA-MB-435 and MDA-MB-231 cell lines) and negative controls (MCF-7 cell line). The cell lines used have been well characterised regarding the methylation status of their ER promoter (Lapidus *et al*, 1998). Serial dilutions of the positive control DNA were used to generate a calibration curve for each analysis. To determine the relative levels of methylated ER promoter DNA in each sample, the values of ER promoter methylation were compared with the values of the internal reference, the housekeeping gene *ACTB.* Dilution series showed linearity of amplification down to 1 : 10 000 for rt-MSP for methylated ER promoter as well as for *ACTB*.

## Results

To validate our approach and to confirm the findings by Issa *et al* (1994), we first examined primary invasive CRCs and lymph nodes with histologically proven metastasis from 14 patients with UICC stage III (any pT, pN1–2, M0) CRC. In 14 out of 14 (100%) CRC and in 13 out of 14 (93%) histologically positive lymph nodes, an aberrantly methylated ER promoter sequence was detected. None of the lymph nodes of the negative control group (0 out of 12), resected for non-neoplastic bowel disease (obstruction or ulcerative colitis), showed methylation of the ER promoter. Next, lymph nodes from 49 patients with UICC stage I or II CRC were analysed (Tables 1 and 2). The mean age of the patient population was 67 years (range 36–90), 25 patients were women and 24 men. Eleven patients had UICC stage I disease corresponding to TNM category pT2pN0 and 38 patients had UICC stage II with pT3pN0 (*n*=37) and pT4pN0 (*n*=1) tumours, respectively. In all, 9 (18%) patients had cancer of the rectum, 12 (24%) of the sigmoid colon, 7 (14%) of the transverse and descending and 21 (43%) of the ascending colon, respectively. At the last follow-up as of December 2006, the average follow-up was 64 months (range 47–79). Two patients with rectal cancer had received adjuvant radiochemotherapy.

Ten of 49 patients died during the follow-up period, three from disease recurrence, one from an accident and six from cardiovascular disease. In total, four patients developed recurrence (overall recurrence rate 8.2%). One patient developed local and distant recurrence, one patient developed solely local recurrence and two patients developed solely distant metastases. Patients who died and did not develop tumour recurrence were treated as censored by statistical survival analysis.

Both patients with local recurrence were positive by rt-MSP (sensitivity 100%, positive predictive value 13.3%). They had well and moderately differentiated tumours of the rectum and sigmoid, respectively, and both had been staged as pT3 pN0 cM0 (UICC stage II). In both patients with local recurrence, six and seven lymph nodes had been examined. Local recurrence was diagnosed 11 and 33 months after initial diagnosis. Both patients died of their tumour recurrence.

Of the 49 patients tested, 15 (31%) had ER methylation-positive lymph nodes. Thirteen of those (86%) remained disease free and two (14%) developed local recurrence. In the resected lymph nodes of 34 of the 49 patients (69%), no ER promoter methylation could be detected and none of these patients experienced a local relapse. Of the 13 patients with positive ER promoter methylation in their resected lymph nodes and a disease-free follow-up, three had CRC UICC stage I and 10 UICC stage II. Regarding age, gender, tumour localisation, UICC stage, grading and the number of resected lymph nodes, there were no statistically significant differences between the patients with and without positive lymph nodes detected by ER promoter methylation.

None of the rt-MSP-negative patients (0 out of 34) developed local recurrence (specificity 72.3%, negative predictive value 100%), but 2 out of 34 (6%) rt-MSP-negative patients developed metastases in the liver and lung without evidence for lymph node or local recurrence. In these patients there was no methylation of the ER promoter in the lymph nodes removed at surgery. In 27 out of the 49 patients with CRC UICC stage I/II, MSI status, as previously assessed (Gerlach *et al*, 2006), was correlated with rt-MSP results and other clinical variables (Table 2); 7 out of 27 patients were MSI+. There was no statistical correlation between microsatellite status, rt-MSP results and local or distant recurrence or any other clinical variables.

## Discussion

According to clinical staging systems (UICC, Dukes and Astler-Coller), the presence of lymph node metastases at the time of diagnosis predicts a poor prognosis for CRC patients. But even among patients with lymph nodes negative by histopathologic criteria (pN0), 10–20% will develop local tumour recurrence, which has been attributed to micrometastases outside the resected lymphatic drainage area. The most sensitive methods to detect micrometastases are immunohistochemistry (IHC) and nucleic acid-based methods. Only few studies could show micrometastases detected by IHC in lymph nodes of UICC stage I and II patients to correlate with disease recurrence and survival (Noura *et al*, 2002a, 2002b; Lee *et al*, 2006; Messerini *et al*, 2006). These conflicting data may be due to the specificity and sensitivity of proteins used for detection as well as the variable thickness and number of tissue sections analysed (Nordgard *et al*, 2003). Therefore, we did not compare the results of rt-MSP with IHC studies.

In addition, there have been several reports on the detection of CK mRNA by RT–PCR. Some studies showed a correlation between CK 20 expression detected by RT–PCR and disease recurrence as well as overall survival, whereas other studies did not (Ichikawa *et al*, 1998; Liefers *et al*, 1998; Sanchez-Cespedes *et al*, 1999; Bernini *et al*, 2000; Miyake *et al*, 2001; Noura *et al*, 2002a, 2002b; Merrie *et al*, 2003; Ho *et al*, 2004; Lassmann *et al*, 2004; Pellise *et al*, 2004; Lee *et al*, 2006). As CK expression is also found in granulocytes and normal blood cells, the highly sensitive RT–PCR may produce false-positive results as described by Bostick *et al* (1998), Jung *et al* (1999) and Bustin *et al* (2000). This hypothesis is supported by a report by Rosenberg *et al* (2002)*,* who found CK-positive cells on histological examination exclusively on the outside of lymph nodes in almost 30% of RT–PCR-positive specimens. Other markers, including CEA, MUC2, GCC, matrilysin, *β*-HCG and others, have been explored to detect lymph node micrometastases by RT–PCR and yielded inconsistent results (Ichikawa *et al*, 1998; Cagir *et al*, 1999; Bernini *et al*, 2000). Expression levels of these different markers can vary considerably among tumour specimens of the same entity. Therefore, these markers have to be compared with the primary tumour (Hanski *et al*, 1997; Waldman *et al*, 1998). In contrast, the ER promoter methylation is present in almost 100% of CRC. Also, in our population, all primary CRCs were positive for ER promoter methylation. Next, we were able to show ER promoter methylation as a good marker of lymph node metastases. To use this marker for the detection of micrometastases we analysed lymph nodes from a study population with UICC stage I and II CRCs. Instead of expression levels here we detected gene promoter methylation by PCR-based methods. The high stability of DNA compared with RNA is one of the main advantages of DNA-based detection methods, which therefore can be applied to the analysis of paraffin-embedded tissues. We demonstrated rt-MSP of DNA from paraffin-embedded lymph node tissue as a robust and practicable method to detect micrometastases.

The high stability of DNA compared with RNA is one of the main advantages of DNA-based detection methods, which therefore can be applied to the analysis of paraffin-embedded tissues. We demonstrated rt-MSP of DNA from paraffin-embedded lymph node tissue as a robust and practicable method to detect micrometastases.

The high negative predictive value was achieved, because none of the 33 patients negative for ER promoter methylation in their lymph nodes developed local recurrence. On the other hand, not all MSP-positive patients developed local recurrence during follow-up. This limitation might in part be explained by a too short follow-up period, the heterogeneity of the study population in terms of the number of investigated lymph nodes or other molecular factors, respectively. The small number of recurrences is another limitation that does not allow a statistical multivariable analysis and results in a low positive predictive value.

Molecular factors have recently been shown to predict disease outcome as distinct genetic subtypes of CRC are defined (Shen *et al*, 2007). Especially, MSI status has been correlated with recurrence and prognosis of CRC (Gryfe *et al*, 2000; Kim *et al*, 2007). Therefore, we compared the MSI status of primary tumours in a subset of patients with the presence of ER promoter methylation in lymph nodes and disease recurrence. Of 27 patients, seven were MSI+, but showed no clear-cut trend for rt-MST results and disease recurrence. On account of the small number of recurrences and the limited statistical significance we did not compare molecular variables other than MSI.

Another reason for the high rate of detected micrometastases by rt-MSP compared with recurrences might be the high sensitivity of rt-MSP, which may detect single tumour cells in a lymph node that might not have the potential to develop to a clinically relevant tumour. This obviously raises the question of the clinical significance of micrometastases for the individual patient. However, the most likely explanation for the lower positive predictive value seems to lie in a potentially complete resection of all micrometastases bearing lymph nodes at surgery.

Lymph node and distant metastases represent two different routes of CRC progression. Whereas the first is probably due to a spread of tumour cells through the local lymph system, the latter arises from circulating tumour cells. Histopathological lymph node involvement is a predictor of disease recurrence. Generally, the route of tumour spread is dependant on the overall tumour load. This interpretation is supported by the close correlation between the quantitative level of CEA transcripts in serum and tumour stage (Ho *et al*, 2004; Ohlsson *et al*, 2006). For local recurrence, ER promoter methylation in lymph nodes could narrow down the patients at risk but not for distant metastases. Overall, the correlation of micrometastatic lymph node involvement together with tumour DNA in plasma from CRC patients may better assess the overall risk of disease recurrence.

In summary, rt-MSP for ER promoter methylation may represent a sensitive and robust method to identify patients with UICC stage I and II CRC at risk for local recurrence. Positive patients have a risk for local recurrence similar to UICC stage III.

## Figures and Tables

**Table 1:** Summary of patient characteristics

**Patient characteristic**	***n*=49**	
Age mean (range)	67	range 36–90
Male	24	49%
Female	25	51%
		
*UICC stage*		
I	11	22%
II	38	78%
		
*pT category*		
pT2	11	22%
pT3	37	76%
pT4	1	2%
		
*Grading*		
Well differentiated	3	6%
Moderately differentiated	43	88%
Poorly differentiated	3	6%
		
*Tumour site*		
Ascending colon	21	43%
Transverse colon	4	8%
Descending colon	3	6%
Sigmoid colon	12	24%
Rectum	9	18%
		
*MSI status*		
Positive	7	26%
Negative	20	74%

MSI=microsatellite instability; UICC=International Union Against Cancer.

**Table 2:** Case-specific clinicopathological parameters and experimental data on the number of investigated lymph nodes and corresponding rt-MSP results (*n*=49)

**Patient**	**pT category**	**Grading**	**MSI+**	**Recurrence**	**Number of investigated lymph nodes**	**rt-MSP**
1	3	2	Yes	No	11	−
2	3	2	No	No	18	+
3	3	2	No	No	29	+
4	3	2	No	Local	6	+
5	3	2	ND	No	24	−
6	2	2	No	No	5	−
7	3	2	ND	No	7	−
8	3	2	No	Distant	14	−
9	3	2	ND	No	5	−
10	2	1	ND	No	9	−
11	3	2	No	No	5	+
12	2	2	Yes	No	9	−
13	3	2	No	No	4	−
14	2	2	No	No	11	−
15	3	3	No	No	16	−
16	3	2	ND	No	4	−
17	4	2	Yes	No	17	+
18	3	2	ND	No	1	−
19	3	2	ND	No	3	−
20	3	2	No	No	10	−
21	3	2	ND	No	7	−
22	3	2	No	No	9	−
23	2	2	Yes	Distant	4	−
24	3	2	ND	No	2	−
25	2	2	ND	No	2	−
26	3	2	No	No	5	+
27	3	2	ND	No	4	−
28	2	2	Yes	No	19	+
29	3	2	ND	No	7	−
30	3	2	ND	No	4	+
31	3	1	Yes	Local and distant	7	+
32	3	2	ND	No	5	−
33	3	2	No	No	5	+
34	3	3	No	No	7	+
35	2	2	No	No	10	+
36	3	2	Yes	No	17	−
37	3	2	No	No	14	−
38	3	1	No	No	6	+
39	3	2	ND	No	21	−
40	3	2	ND	No	12	−
41	3	2	ND	No	18	−
42	3	2	No	No	6	−
43	2	3	ND	No	13	−
44	2	2	No	No	9	+
45	3	2	ND	No	5	−
46	2	2	ND	No	16	−
47	2	2	ND	No	22	−
48	3	2	ND	No	14	−
49	3	2	No	No	9	+

MSI+=microsatellite instability; ND=not determined; rt-MSP=real-time methylation-specific PCR.
